# Parotid gland, an exceptional localization of sebaceous carcinoma: case report

**DOI:** 10.1186/s12907-016-0031-y

**Published:** 2016-06-08

**Authors:** Mouna Khmou, Karima Laadam, Nadia Cherradi

**Affiliations:** Department of Pathology, Hospital of Specialities, Rabat, Morocco; Faculty of Medicine and Pharmacy Rabat, University Mohammed V Rabat, Rabat, Morocco

**Keywords:** Parotid, Gland, Sebaceous, Carcinoma, Rare

## Abstract

**Background:**

Sebaceous carcinoma (SC) is a rare malignancy, occurring predominantly in eyelids. Till date, only 25 cases of sebaceous carcinoma (SC) of the parotid gland have been reported in world literature.

**Case presentation:**

A 33 year-old male presented with left sided laterocervical mass. Clinical examination showed enlargement of the left parotid gland, with cervical lymphadenopathy. No skin lesions were found. A resection of the gland was performed. Pathological findings were consistent with primary sebaceous carcinoma of the parotid gland.

**Conclusion:**

Sebaceous carcinoma of the parotid gland is extremely uncommon. Clinical and radiological features are not specific. The aim of this report, is to describe histopathological, and immunohistochemical findings of this rare entity, and discuss differential diagnosis.

## Background

Sebaceous glands are holocrine adnexal components of the skin, usually found in close association with hair follicles [1]. Sebaceous tumors are uncommon, and their classification is controversial [2] Predominantly occurs in eyelids [3], other sites may exceptionally be involved. In the English literature, only 25 cases of sebaceous carcinoma (SC) of the parotid gland have been reported [4]. Sebaceous carcinoma is defined by the WHO as “a malignant tumor composed of sebaceous cells of varying maturity that are arranged in sheets and/or nests with different degrees of pleomorphism, nuclear atypia, and invasiveness” [5]. Diagnosis may be difficult, given the low incidence and inconsistencies in histopathologic classification. Regardless of the location, sebaceous carcinomas must be considered as an aggressive neoplasm with a potential for regional and distant metastasis [2].

We report an additional case, discuss the clinical and pathologic features ; and briefly review of the literature,

## Case presentation

A 33 year-old Moroccan male presented with left sided laterocervical mass, which had persisted for four months. No personal or family history was noted. He had no previous history of smoking, alcohol use, or irradiation. The mass had slowly grown with occasional pain. He had no fever, chills, or weight loss. Upon physical examination, the left parotid gland was enlarged, firm, with cervical lymphadenopathy, no skin lesions were found. Ultrasonography and computed tomography revealed a solid mass involving the parotid gland. A biopsy revealed a poorly differentiated carcinoma.

**Fig. 1:**
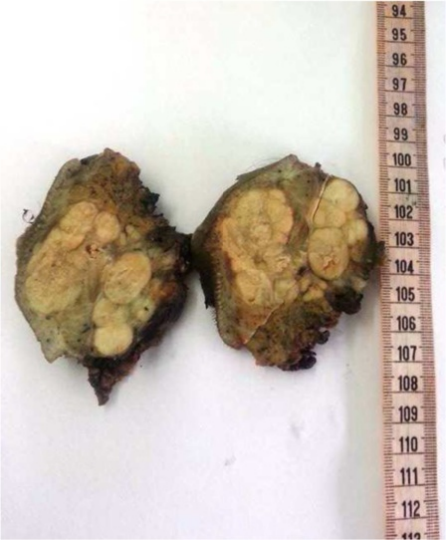
Macroscopic aspects of the tumor after the en-block removal

The patient underwent tumor excision. The excised mass measuring 21,5 × 9 × 6 cm, with skin tag measuring 11 × 10 cm. The cut surface of the tumor was firm tan-gray, lobulated, measuring 6 × 5,5 × 5 cm, with, apparently normal looking, salivary gland tissue at the peripheral margin (Fig. 1). Meticulous and extensive sampling of the tumor was done.

**Fig. 2:**
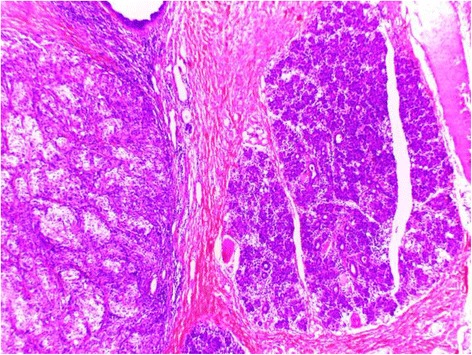
Low magnification of the tumor within to the parotid parenchyma

**Fig. 3:**
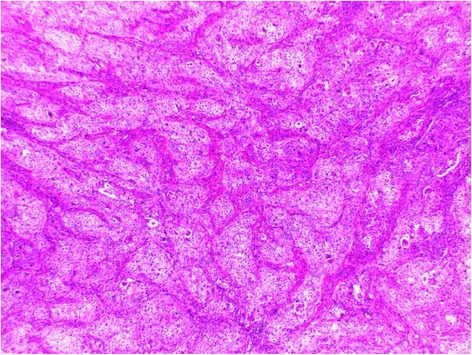
The tumor lobules composed of large foamy cells surrounded by basaloid cells

**Fig. 4:**
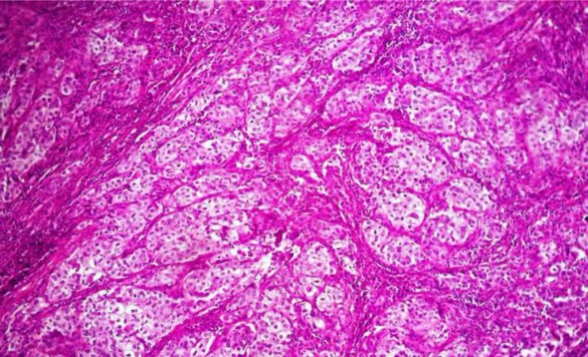
numerous cells with sebaceous differentiation

**Fig. 5:**
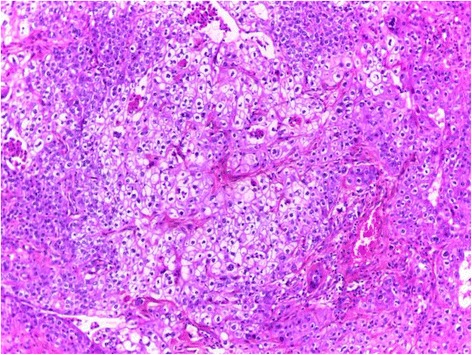
Tumor cells showing nuclear atypia and mitosis

Histopathological examination revealed a lobulated tumor with expansive growth within parotid parenchyma (Fig. 2). It was composed of nests of two cell populations : large foamy cells with centrally located nuclei and vacuolated clear cytoplasm, surrounded by closely packed smaller basaloid cells with scanty cytoplasm (Fig. 3). Large tumor cells showed sebaceous differentiation (Fig. 4), with cellular pleomorphism, high mitotic activity (Fig. 5) and necrosis. Some areas showed squamous islands with keratin pearl formation. Periodic acid–Schiff (PAS) was negative in the foamy, large cells.

**Fig. 6:**
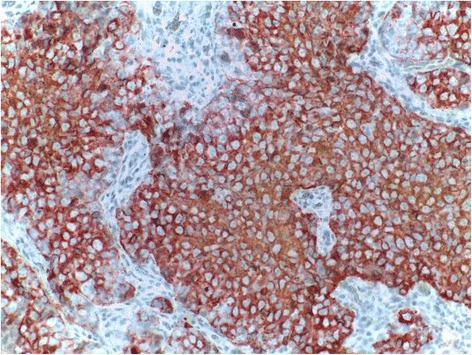
Immunohistochemistry shows positive staining for EMA

Immunohistochemical staining of the tumor showed expression of epithelial membrane antigen (EMA) (Fig. 6), pancytokeratin, and p63 in all neoplastic cells, and focaly B-Catenin. They lacked expression of CK5/6, CEA, S100, CD10, Vimentin, melan A, and CD45. The diagnosis of Sebaceous carcinoma of the parotid gland was made.

**Fig. 7:**
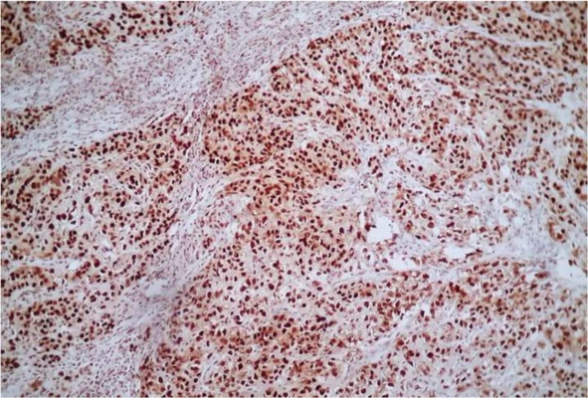
Immunohistochemistry shows positive staining for MSH2 in tumor cells and lymphocytes

**Fig. 8:**
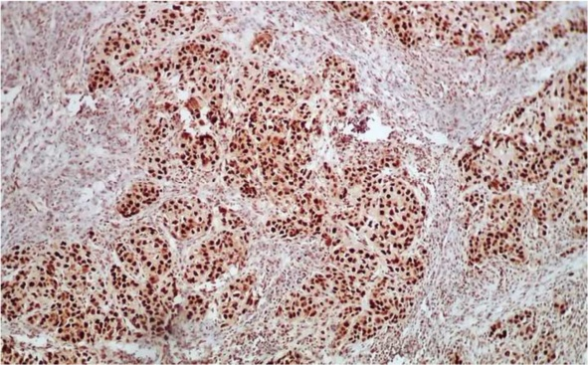
Immunohistochemistry shows positive staining for MLH1 in tumor cells

Since a recent literature review report a relation between sebaceous carcinoma and MSH2 mutation, we evaluated by immunohistochemistry MLH1 and MSH2 protein expression. Strong nuclear expression of both proteins was found (Figs. 7 and 8). All surgical margins were microscopically negative. A staging computerised tomography (CT), gastrointestinal endoscopy and colonoscopy were preformed and no tumor was found. Thus, the Muir-Torre syndrome was excluded. Adjuvant radiotherapy was decided. The patient is alive without signs of tumor recurrence after 1 year of follow-up.

## Discussion

Sebaceous carcinoma was first described in the salivary glands by Rauch and Masshoff in [6]. It is a rare and aggressive malignant neoplasm usually occurring in the head and neck region [3], involving in 75 % the periocular region, particularly the upper eyelid in elderly women [2]. Only handful cases of primary salivary sebaceous carcinoma had been described, most of them involving the parotid gland, rarely the submandibular and minor salivary glands [7].

The histogenesis of sebaceous carcinoma in the parotid gland remain unclear. Sebaceous differentiation of salivary ducts is seen in both normal and chronic sialadenitis [3]. The parotid gland in the present case had mild chronic inflammation. The current hypothesis is that sebaceous carcinoma arises from pluripotent stem cells, which can differentiate into sebaceous cells [7]. It is accepted that sebaceous lymphadenocarcinoma arises from sebaceous lymphadenoma, but SC of the salivary glands seems to be a de-novo lesion [2]. SC can be part of Muir-Torre syndrome (MTS), and it was suggested that expression of retinoid X receptor beta and gamma could be related to the development of SC [8]. Muir-Torre syndrome is a phenotypic variant of hereditary non-polyposis colorectal cancer (HNPCC) or Lynch syndrome. Germline mutation in hMSH2 and hMLH1 genes are often associated with this disorder [9]. The result for DNA mismatch repair genes in sporadic sebaceous carcinoma is inconclusive [3]. The most common site for sebaceous neoplasms in Muir Torre Syndrome is the eyelids and nose, and after extensive review of the literature, the association between parotid sebaceous carcinoma in Muir Torre Syndrome has been reported only once. In this present case, no association with Muir-Torre syndrome was established, and immunohistochemical staining showed normal nuclear expression of MLH1 and MSH2 in tumor cells.

SC in the parotid gland is reported to occur in both genders with the same incidence, and may have an increased frequency in the asian population [2]. This tumor has a bimodal age distribution, with a peak in the second decade and another one in the seventh decade of life (with a range of 6–92 years) [4].

Clinically, the duration of symptoms is highly variable and ranges from few months to 20 years. SC typically present as slowgrowing swellings with variable pain, facial nerve involvement, and fixation to the overlying skin. Rare cases have arisen from a preexisting pleomorphic adenoma [10]. Our patient has no history of an untreated or recurrent pleomorphic adenoma ; also an extensive sampling of the tumor was done, and no area of residual benign mixed tumour, was found.

Grossly, tumors range in size from 0.6 to 8.5 cm, frequently appear to be well circumscribed or partially encapsulated [5], gray to tan on the cut surface [11]. Microscopically, the tumor consists of sheets, nests, or cords with expansive growth. Duct-like structures may be numerous and cystic spaces of varying sizes are occasionally present. The tumor may exhibit, pleomorphic cells with variable degrees of cytologic atypia [11]. In well-differentiated tumors, the cells have hyperchromatic nuclei and abundant, cytoplasmic foamy vacuolization, giving a typical sebaceous appearance [5]. Typically, sebaceous neoplasic cells are located in the central parts of the nests, which peripherally show more undifferentiated cells with scarcer cytoplasm. A transition is observed between sebaceous and undifferentiated cells [12]. Squamous differentiation in sebaceous neoplasms is common [3]. Scattered mucous cells, xanthogranulomatous reaction and oncocytic metaplasia are occasional findings [11]. A positive lipid stain, such as oil-red-O or Sudan IV, is helpful for establishing the diagnosis [1], but in most cases not possible because frozen sections are not always available [3].

Immunohistochemically, Androgen receptor (AR) is useful in the diagnosis of poorly differentiated sebaceous carcinomas [3], but there are no studies of AR in SC of the salivary glands [2]. On the contrary, SC of the breast is known to be positive for AR, indicating that expression of this receptor may be related to the site of tumor origin [2]. EMA and HMFG1 (human milk fat globule1) are expressed mainly by the sebaceous cells both in the cytoplasm and membrane, but are negative in most of the basaloid peripheral cells [2]. Several case reports and case series have confirmed the usefulness of immunohistochemistry in diagnosing SC [4]. But since most reported cases have no extensive information on this issue, further studies are needed to determine the most useful immunohistochemistry panel in the diagnosis of SC.

Sebaceous carcinoma must be distinguished from mucoepidemoid carcinoma, poorly differentiated squamous carcinoma, basal cell carcinoma, and metastatic clear cell renal carcinoma [4].

Unlike mucoepidemoid carcinoma, PAS and D-PAS in SC stains negative. Malignant squamous cells may accumulate glycogen and demonstrate clear cytoplasm. Which can be confirmed by PAS staining, and positivity of CK5/6 on immunohistochemistry.

The lack of lymphoid tissue did not support a diagnosis of sebaceous lymphadenocarcinoma [9].

Sebaceous Epithelial-Myoepithelial Carcinoma (EMC) must be considered as a differential diagnosis. This tumor is composed by bilayered ductal structures composed of inner epithelial-type cells and outer myoepithelial cells with clear cytoplasmic. The key feature to distinguish sebaceous EMC from sebaceous carcinoma is to reveal the myoepithelial nature of the tumor cells. Mostly by using myoepithelial markers, such as calponin, a-SMA, MSA, p63, CK 14, S-100 protein, and vimentin, on immunohistochemistry [13].

The treatment of choice is wide surgical excision. Parotidectomy, extended parotidectomy, and/or neck dissection maybe required to achieve complete resection [4]. Postoperative radiotherapy and chemotherapy, in tumors with a high microscopic grade or clinical stage, has occasionally been proposed [5, 9]. Out of reported cases, 9 were treated with radiotherapy. Although most reported cases have no information on the tumor pro-gression only 1 case treated with radiotherapy recurred [4]. This indicates the beneficial role of radiotherapy as treatment option in SC of the parotid. Our patient has no signs of tumor recurrence after 1 year after adjuvant radiotherapy. Metastasis may occur in the lung, brain, and regional lymph nodes [4].

There are too few reported cases to make accurate prognostic statements. Although extraocular cases were considered less aggressive, this is no longer accepted [2]. At least 6 cases of SC of the salivary glands have been described with recurrence and metastasis [12].

## Conclusion

In summary, primary sebaceous carcinoma of the salivary glands is extremely rare and aggressive tumor, and because of its rarity, clinicopathological characteristics and histogenesis are not fully understood.

## Abbreviations

SC, Sebaceous carcinoma; PAS, periodic acid–Schiff; EMA, epithelial membrane antigen; MTS, Muir-Torre syndrome; HMFG1, human milk fat globule1; EMC, Epithelial-Myoepithelial Carcinoma
